# Physician-Modified Endografts for Complex Aortic Aneurysms in Japan: Current Status, Clinical Outcomes, and Guideline Integration

**DOI:** 10.3400/avd.ra.25-00095

**Published:** 2026-02-07

**Authors:** Tsuyoshi Shibata, Yutaka Iba, Shingo Tsushima, Tomohiro Nakajima, Junji Nakazawa, Ayaka Arihara, Kenichi Kato, Shigeki Komatsu, Masato Yonemori, Kenta Yoshikawa, Shun Hayasaka, Hirokazu Sugiura, Hajime Maeda, Nobuyoshi Kawaharada

**Affiliations:** 1 Division of Cardiovascular Surgery, Department of Surgery, Sapporo Medical University, Sapporo, Hokkaido, Japan; 2 Division of Radiology and Nuclear Medicine, Sapporo Medical University, Sapporo, Hokkaido, Japan; 3 Department of Rehabilitation, Sapporo City General Hospital, Sapporo, Hokkaido, Japan

**Keywords:** physician-modified endograft, aortic aneurysm, endovascular aortic repair, guideline, Japan, complex aortic aneurysm

## Abstract

In Japan, the absence of commercially available fenestrated and/or branched endografts has necessitated widespread adoption of physician-modified endografts (PMEGs) for complex aortic aneurysms. This paper compares PMEG use in Western countries and Japan, summarizes multicenter outcome data, and highlights the gap between real-world practice and current Japanese aortic disease guidelines. Recent Japanese series report high technical success and acceptable mid-term outcomes, comparable to Western reports. While long-term durability remains uncertain, structured training, national registries, and standardized protocols are essential. Guideline acknowledgment of PMEGs could improve safety, consistency, and international alignment in complex endovascular therapy. Establishing structured training, national registries, and evidence-based policy recognition of PMEGs is essential to ensure safe and standardized practice in Japan.

## Introduction

Endovascular aortic repair (EVAR) has become the standard treatment for abdominal aortic aneurysms in appropriately selected patients.^1–4)^ However, for complex aneurysms involving visceral arteries—such as pararenal, juxtarenal, and thoracoabdominal aortic aneurysms—standard EVAR is often insufficient. In such cases, fenestrated and/or branched endovascular aortic repair (F/BEVAR), using custom-made devices (CMDs) or off-the-shelf devices (OSDs), is generally recommended in Western countries, with favorable outcomes reported in large registries and multicenter studies.^3–5)^

In Japan, the clinical availability of company-manufactured F/BEVAR devices remains extremely limited due to regulatory and logistical constraints. Consequently, physician-modified endografts (PMEGs)—in which operators intraoperatively modify commercially available stent grafts to accommodate visceral branches—have emerged as a practical and, in some cases, indispensable solution.^6)^ Over the past decade, several Japanese centers have developed substantial expertise in PMEG techniques, often incorporating 3-dimensional (3D) modeling and meticulous preoperative planning under the oversight of institutional review boards and while addressing ethical considerations.^7)^ Multicenter observational studies have reported encouraging technical success rates and mid-term clinical outcomes, even in high-risk patients with complex anatomy.

Despite this, the current Japanese aortic disease guidelines (JCS/JSCVS/JATS/JSVS 2020) make no mention of PMEGs, creating a discrepancy between real-world practice and formal recommendations.^2)^ While the safety and long-term durability of PMEGs have yet to be definitively established, their widespread use across Japan and the growing body of literature underscore their clinical relevance. Given their role in addressing unmet treatment needs under current regulatory constraints, it is reasonable to argue that PMEGs should be acknowledged—at least descriptively—in future iterations of the national guidelines.

This article reviews the current status of PMEG use in both Western countries and Japan, summarizes supporting clinical data, and discusses considerations for their potential inclusion in future Japanese aortic treatment guidelines.

## PMEG Experience in Western Practice

In Western countries, the treatment of complex aortic aneurysms involving visceral branches has evolved substantially over the past 2 decades, largely driven by the widespread adoption of F/BEVAR.^8–10)^ Company-manufactured F/BEVAR devices such as the Cook Zenith fenestrated and the off-the-shelf multibranched T-Branch stent graft (Cook Medical, Bloomington, IN, USA) are available as CMDs or OSDs and have obtained regulatory approval in the United States, Europe, and Australia.

In the United States, the Food and Drug Administration permits the use of both custom-made fenestrated and branched endografts under the Investigational Device Exemption framework, with increasing application in both elective and urgent settings. Similarly, CE (Conformité Européenne)-marked devices are widely available across Europe.

Importantly, both European and North American guidelines recognize F/BEVAR as a valid treatment option for juxtarenal, pararenal, and thoracoabdominal aneurysms in patients at high surgical risk. The 2024 ESVS guidelines on the management of abdominal aortic aneurysms state that “fenestrated or branched endografts may be considered for patients with complex aortic anatomy, particularly when open repair carries excessive risk.”^3)^ Likewise, the AHA clinical practice guidelines support the use of F/BEVAR for high-risk patients, with growing emphasis on individualized, anatomy-specific device planning.^4)^

While PMEGs are not considered a first-line strategy in Western countries where commercial devices are readily available, they have been increasingly used under specific institutional protocols or in urgent cases when CMDs are not feasible due to time constraints. Several high-volume centers have published encouraging data on PMEG feasibility and safety,^11–14)^ contributing to international procedural standardization and training initiatives. Notably, the latest ESVS guidelines recommend considering PMEGs in urgent situations when no commercial device is available.^3)^

This global landscape stands in stark contrast to the situation in Japan, where the absence of approved commercial devices necessitates broader reliance on PMEGs. Recognizing PMEGs in the Japanese guideline context would therefore not only be clinically relevant but also align with evolving international endovascular strategies.

## Current Status of PMEGs in Japan: Clinical Practice and Guideline Gaps

Unlike Western countries where company-manufactured F/BEVAR devices are available, Japan has no regulatory approval for fenestrated and/or branched endografts, creating a significant gap in endovascular options for complex aortic aneurysms. In this context, PMEGs have become an essential therapeutic modality for managing pararenal and thoracoabdominal aneurysms in high-risk patients.

Several leading Japanese institutions have implemented standardized PMEG protocols, drawing on international experience while adapting them to locally available devices. These centers have developed expertise in device modification with fenestrations and inner branches to preserve visceral perfusion, frequently employing 3D-printed models or advanced preoperative planning to ensure precise customization.^7,15,16)^

Despite their clinical indispensability, PMEGs are absent from the current Japanese aortic disease guidelines, which focus predominantly on open surgery and do not address endovascular repair for complex anatomy unsuitable for standard EVAR.^2)^ This omission poses challenges for institutional policy-making, insurance coverage, physician education, and dissemination of best practices.

As Japan faces an aging population and increasing case complexity, the formal recognition of PMEGs in national guidelines will be an urgent priority. Such recognition would reflect real-world practice and align Japan’s endovascular strategies with international standards, regardless of the recommendation grade.

## Discussion and Future Directions

Recent international and Japanese data provide important context for the outcomes summarized in **Table 1**.^6,11–14,16)^ Chait et al.^11)^ reported 5-year results of 156 PMEG cases, demonstrating acceptable long-term durability with 91% primary target-artery patency and higher complication rates in thoracoabdominal aneurysms than in complex abdominal aneurysms. Starnes et al.^12)^ presented 12-year outcomes from their IDE (Investigational Device Exemption) clinical trial of 203 PMEG implants, showing durable branch preservation, a 93.7% technical success rate, an 82.6% treatment success rate at 12 months, and very low late rupture rates. Tsilimparis et al.^13)^ published the largest international dataset to date—1274 cases across 19 centers—reporting 94% technical success, 5.8% 30-day mortality, and excellent long-term target-vessel patency. Sanders et al.^14)^ summarized over a decade of institutional PMEG experience, noting progressive reductions in fluoroscopy time and completion of endoleaks, with perioperative and long-term outcomes comparable to commercial ZFEN devices despite greater procedural complexity. In Japan, Shibata et al.^6)^ demonstrated high per-vessel technical success (98.5%) and acceptable in-hospital mortality in a multicenter cohort of 121 high-risk patients, while Shibata et al.^16)^ highlighted the feasibility of physician-modified fenestrated/inner-branched repair, even in urgent settings, despite anatomical complexity. This overview allows positioning the present study within the context of the evolving international experience. This paper highlights the unique role of PMEGs in Japan, where the absence of company-manufactured F/BEVAR devices has created a distinct clinical environment compared with Western countries. In the United States, Europe, and Australia, F/BEVAR with CMDs or OSDs is integrated into mainstream practice, supported by regulatory approval, established manufacturing, and extensive registry data.^3–5)^ In contrast, Japanese physicians have adopted PMEGs out of necessity rather than preference. Technical success rates and mid-term outcomes from high-volume Japanese centers are encouraging and align with Western PMEG and CMD series.^6)^ However, these findings should be interpreted with caution, as most evidence comes from retrospective observational studies in experienced institutions, with inherent selection bias and limited generalizability. Moreover, the long-term durability, reproducibility across operators, and comparative effectiveness versus open surgical or commercial endovascular options remain insufficiently defined.

**Table 1:** Summary of representative reports on physician-modified endografts

First author (year, journal)	Country	Patients (n)	Type of modification	Technical success per patient (%)	In hospital/30-day mortality (%)	Major adverse event** (study-defined, %)
Chait^11)^ (2023, J Vasc Surg)	USA (single center)	156 (mean age: 75) CAAA 89, TAAA 67	Fenestrated, scallop, directional branched	96	6 (30-day) (CAAA 2, TAAA 10)	26
Starnes^12)^ (2024, Ann Surg)	USA (single center)	203 (mean age: 75.6) Juxta 203	Fenestrated	93.7	2.9 (30-day)	15
Tsilimparis^13)^ (2024, Circulation)	International (19 centers)	1274 (mean age: 74) CAAA 582, TAAA 692	Fenestrated 83.1%, branched 3.6%, combo 13.4%	94	5.8 (30-day) (CAAA 4.1, TAAA 7.2)	25.2
Shibata^6)^ (2024, Interdiscip Cardiovasc Thorac Surg)	Japan (10 centers)	121 (mean age: 75.6) PRAA 62, TAAA 59	Fenestrated base	PRAAs: 93.5 TAAAs: 98.3	5.8 (perioperative*) (PRAA 3.2, TAAA 8.5)	17.4 (PRAA 11.3, TAAA 23.7)
Shibata^16)^ (2024, Eur J Cardiothorac Surg)	Japan (6 centers)	38 (mean age: 80.5) PRAA 7, TAAA 31	Fenestrated/inner branched	89.5	15.8 (in hospital) (elective 9.7, non-elective 42.9)	26.3
Sanders^14)^ (2025, J Vasc Surg)	USA (single center)	184 (mean age: 76) Juxta 122, Supra 20, TAAA 42	Fenestrated/branched	Not reported	4.9 (perioperative*)	MI 4.3, dialysis 2.2, respiratory failure 7.1, SCI 8.7

*Perioperative outcomes were assessed within the index admission or within 30 days of the index procedure, whichever was longer.

**Definitions were broadly similar across studies (death, MI, stroke, renal failure/dialysis, respiratory failure, bowel ischemia, paraplegia). The study by Starnes additionally included procedural blood loss ≥1000 mL.

CAAA: complex abdominal aortic aneurysms; Juxta: juxtarenal abdominal aortic aneurysm; MI: myocardial infarction; PRAA: pararenal abdominal aortic aneurysms; SCI: spinal cord ischemia; Supra: suprarenal abdominal aortic aneurysm; TAAA: thoracoabdominal aortic aneurysms

These uncertainties explain why PMEGs cannot currently be regarded as a broadly recommended treatment strategy. Their use should be limited to carefully selected patients in specialized, high-volume centers with multidisciplinary expertise and rigorous perioperative protocols. However, the complete absence of PMEGs from the current Japanese aortic disease guidelines creates a gap between formal recommendations and real-world practice. This gap may hinder institutional decision-making, training, and the development of standardized protocols. The aim of including PMEGs in guideline discussions is not to promote indiscriminate use but to acknowledge their role under current Japanese regulatory conditions and to ensure that clinical decisions are informed by the best available evidence. To strengthen alignment between real-world practice and national recommendations, it is important to outline realistic pathways for incorporating PMEG-related evidence into future Japanese guideline revisions. In Japan, aortic disease guidelines are updated through multidisciplinary working groups organized by the Japanese Society for Vascular Surgery and the Japanese Society for Cardiovascular Surgery. Within this structure, establishing a dedicated PMEG working group would allow systematic evaluation of emerging evidence, development of standardized terminology, and consideration of indications that reflect Japan’s unique regulatory environment. High-quality domestic data—particularly from the National Clinical Database (NCD) and prospective multicenter PMEG registries—together with consensus meetings and joint society statements, would form the evidence base required for transparent and feasible guideline integration.

Going forward, key priorities include the establishment of structured training, simulation-based workshops, and inter-institutional proctoring; the development of standardized modification and deployment protocols; and the implementation of national registries for procedural and long-term follow-up data. In addition, several concrete strategies should be pursued to create the necessary infrastructure for future guideline inclusion. These include large-scale outcome research leveraging the NCD to provide robust domestic evidence; collaborative training initiatives with industry partners incorporating cadaveric laboratories, advanced simulation, and virtual imaging platforms; academic society–driven PMEG workshops to disseminate best practices; and publication of structured “how-to” papers to standardize procedural pathways and ensure reproducibility. Health-economic analyses and potential collaboration with international registries may further support social recognition of PMEGs as a viable and necessary option. Collaborative, multicenter research will be essential to generating robust domestic evidence on PMEG safety, efficacy, and cost-effectiveness, thereby supporting informed and balanced guideline inclusion in the future.

This paper is descriptive and reflects current PMEG use in Japan under existing regulatory and device availability constraints. Although favorable short- to mid-term outcomes have been reported from experienced centers, current evidence is largely observational, with inherent selection bias and limited generalizability. Furthermore, the long-term durability, reproducibility, and comparative effectiveness of PMEGs relative to established surgical or endovascular options remain insufficiently defined. Therefore, PMEGs cannot currently be regarded as a broadly recommended treatment strategy, and their use should be confined to carefully selected cases—preferably in specialized high-volume centers under rigorous outcome monitoring. Nevertheless, the complete absence of PMEGs from current guidelines creates a gap between formal recommendations and real-world practice. The aim of this paper is not to advocate PMEG use, but to emphasize the need for discussion on how, and in what form, PMEGs should be described in future guideline revisions.

In parallel, the ethical and regulatory governance required for PMEG use in Japan must be clearly recognized. Because PMEG involves intraoperative modification of commercially manufactured devices, its clinical application requires robust ethical oversight. Under Japanese regulatory practice, off-label device use is permitted at the physician’s discretion when conducted under appropriate governance in accordance with Ministry of Health, Labour and Welfare notifications. Routine PMEG practice therefore necessitates institutional review board approval, detailed written informed consent, and clear institutional accountability. Although this review does not report procedures performed at our institution, outlining these principles underscores the standards of safety and compliance under which PMEGs should be implemented in Japan.

### Limitations

This review is narrative in nature and does not follow a systematic review methodology. As a result, the selection of cited studies may be incomplete, and relevant publications—particularly those published in languages other than English—may not have been captured. Furthermore, much of the currently available evidence on PMEGs is derived from retrospective observational studies conducted in high-volume centers, which introduces inherent selection bias and limits generalizability to broader clinical practice. Therefore, the conclusions drawn in this paper should be interpreted with awareness of these inherent limitations.

## Conclusion

PMEGs have filled a critical treatment gap in Japan, enabling life-saving interventions for patients with complex aortic pathology who would otherwise have limited options. As supporting evidence grows, national policies and guidelines should evolve accordingly. We therefore propose:

- Inclusion of a dedicated section on complex endovascular repair, including PMEGs, in the next edition of the national guidelines.
- Formal recognition of PMEGs as a viable and necessary option for patients with complex anatomy unsuitable for standard EVAR or open repair.
- Promotion of collaborative research to generate robust domestic evidence supporting the safety, efficacy, and cost-effectiveness of PMEGs.

Recognizing and regulating PMEGs will not only ensure procedural safety and consistency but also reaffirm Japan’s position as a global contributor to innovation in complex endovascular therapy. At the same time, Japanese PMEG surgeons should remain committed to continuous efforts in establishing standardized protocols and accumulating evidence, thereby ensuring that the clinical use of PMEGs advances in parallel with rigorous scientific validation.
